# Wavelength-Tunable Vortex Beam Emitter Based on Silicon Micro-Ring with PN Depletion Diode

**DOI:** 10.3390/s22030929

**Published:** 2022-01-25

**Authors:** Ivan V. Stepanov, Denis M. Fatkhiev, Vladimir S. Lyubopytov, Ruslan V. Kutluyarov, Elizaveta P. Grakhova, Niels Neumann, Svetlana N. Khonina, Albert K. Sultanov

**Affiliations:** 1 School of Photonics Engineering and Research Advances (SPhERA), Ufa State Aviation Technical University, 450008 Ufa, Russia; stepanov.iv@ugatu.su (I.V.S.); kutluyarov.rv@ugatu.su (R.V.K.); grakhova.ep@ugatu.su (E.P.G.); sultanov.ah@ugatu.su (A.K.S.); 2 Center for Photonic Science and Engineering, Skolkovo Institute of Science and Technology, 121205 Moscow, Russia; v.lyubopytov@skoltech.ru; 3 Chair of Radio Frequency and Photonics Engineering, TU Dresden, 01062 Dresden, Germany; niels.neumann@tu-dresden.de; 4 Department of Technical Cybernetics, Samara National Research University, 443086 Samara, Russia; khonina@ipsiras.ru; 5 Image Processing Systems Institute Branch of the Federal Scientific Research Center “Crystallography and Photonics” of Russian Academy of Sciences, 443001 Samara, Russia

**Keywords:** orbital angular momentum, vortex beam, microring resonator, pn-depletion diode

## Abstract

Herein we propose a design of a wavelength-tunable integrated vortex beam emitter based on the silicon-on-insulator platform. The emitter is implemented using a PN-depletion diode inside a microring resonator with the emitting hole grating that was used to produce a vortex beam. The resonance wavelengths can be shifted due to the refractive index change associated with the free plasma dispersion effect. Obtained numerical modeling results confirm the efficiency of the proposed approach, providing a resonance wavelength shift while maintaining the required topological charge of the emitted vortex beam. It is known that optical vortices got a lot of attention due to extensive telecommunication and biochemical applications, but also, they have revealed some beneficial use cases in sensors. Flexibility in spectral tuning demonstrated by the proposed device can significantly improve the accuracy of sensors based on fiber Bragg gratings. Moreover, we demonstrate that the proposed device can provide a displacement of the resonance by the value of the free spectral range of the ring resonator, which means the possibility to implement an ultra-fast orbital angular momentum (de)multiplexing or modulation.

## 1. Introduction

Since the unique properties of optical beams carrying orbital angular momentum (OAM), also referred to as optical vortices, have been discovered in [1], the request for further research and development in this field has been growing steadily. This was not unreasonable, as applications of the vortex beams turned out to be interesting in a wide variety of areas. One of their most known use cases is trapping and moving particles with the optical tweezers and spanners [2,3]. Optical tweezers empowered with OAM have demonstrated the manipulation of particles with multiple degrees of freedom, as well as the simultaneous trapping of multiple particles [4,5]. Optical beams carrying OAM, e.g., Bessel beams [6], along with other kinds of structured light [7] have also found their application in such a remarkable topic as quantum communications [8], specifically in higher-dimensional quantum key distribution [9], entanglement swapping [10], and multidimensional entanglement [11].

Another major field for vortex beams is optical communications where OAM is usually considered as an additional degree of freedom for multiplexing. The exponentially growing demand for network traffic [12,13] resulted in the fiber-optic lines utilizing time, wavelength, and polarization division multiplexing, which have almost reached the Shannon limit [13,14]. Therefore, the next step on the way to increase the throughput of fiber transmission lines was the space division multiplexing (SDM) [15,16,17]. SDM technology is based on the use of a degree of freedom determined by the transverse distribution of the electromagnetic (EM) field, that corresponds to multiplexing of spatially separated optical fields in multicore fibers (MCF) or using several linear polarized (LP) modes in few-mode fibers (FMF) [18]. SDM concept can be applied in both fiber-optic [17,18,19,20] and atmospheric [21,22,23] optical communication lines.

A common property of optical modes carrying OAM is the presence of a multiplier $e^{i\ell \varphi }$, where *ℓ* is the azimuthal mode index. These modes are the eigenfunctions of the angular momentum operator and carry the OAM proportional to *ℓ* [1]. As the OAM modes represent a basis of orthogonal functions which can be divided spatially by its order *ℓ*, it is convenient to use them in the SDM approach [19,24]. Moreover, OAM multiplexing can be combined with other multiplexing technologies and multilevel modulation formats for increasing throughput to the Tbit level [20,25].

Finally yet importantly, beams carrying OAM are widely used in sensing. In biochemistry, optical vortices were used to detect the molecules of amino acids, nucleotides, and sugars [26]. Diffraction limit [27] and super resolution [28] imaging was reached with focusing of vortex beams. Another example is a temperature sensor consisting of a fiber Bragg grating (FBG), an optical fiber path used to eliminate errors, and a Gaussian beam, interfering with the OAM beam transmitted through the Bragg grating [29]. The principle of operation of this temperature sensor lies in a combination of the thermo-optical effect and the effect of thermal expansion, which appear in the Bragg grating when the temperature changes and leads to a shift in the central wavelength of the reflected spectrum. In turn, the phase difference between the Gaussian beam and the vortex beam leads to the rotation of their interference diagram. The temperature measurement step corresponds to the rotation of the radiation pattern. A similar method can be used to make highly accurate measurements of microstrains caused by pressure and displacement. It is also important to note the recent successes in plasmonic vortex studies: nanometrology approaches [30], generation of the high order plasmonic vortices [31], and OAM-SPR (surface plasmon resonance) based refractive index sensing [32] are making a breakthrough.

In this paper, we propose and numerically verify a novel scheme of real-time OAM order switch for radiated optical vortex beam using a pn-depletion diode integrated into the ring waveguide. The most common solution that can be used to excite optical beams with a helical phase front, and which we employed in the proposed design, is a micro-ring resonator [33]. Usually, this is a ring-shaped waveguide with grating elements for the light beam emission, and a bus waveguide located at a small gap, which couples an input beam into the resonator. Such µm-scale structure was first demonstrated in [34]. Such devices are capable to emit vector optical vortices with definite and quantized OAM states. It is possible due to the ring (or disk) resonators supporting whispering gallery modes (WGM), which carry high orders of OAM. The grating provides a periodic modulation of an effective refractive index, and its working principle is analogous to the operation principle of grating couplers in straight waveguides. The light wave is scattered by the grating elements, and as a result, a part of the radiated power is deflected in the direction of the constructive interference. Because the waveguide has a ring shape and supports WGM, according to the Huygens principle the wavefront of the emitted light should point to the azimuthal direction φ and be helical.

To our knowledge, there has been one system demonstrated based on a single ring resonator that realizes real-time OAM switching. In [35], authors developed an approach to a fast electrically-controlled vortex order switch and demonstrated a scheme with the switching time of down to 20 µs. In this scheme, heaters are used to change the refractive index of the waveguide, and as a result, the effective WGM index changes, which causes the restructuring of the emitted OAM mode. In our case, an inversely-biased pn-junction offers more energy-efficient and faster switching [36] compared to the existing schemes. Therefore, we believe, that this scheme can be used also as a fast (GHz-scale) electro-optical OAM modulator.

The paper is organized as follows: in Section 2 we describe the working principle of the proposed scheme; in Section 3 the device modeling results are presented; and in Section 4 the methodology and the results of analysis of the emitted beams propagation are described.

## 2. Principle of Operation

**Figure 1:**
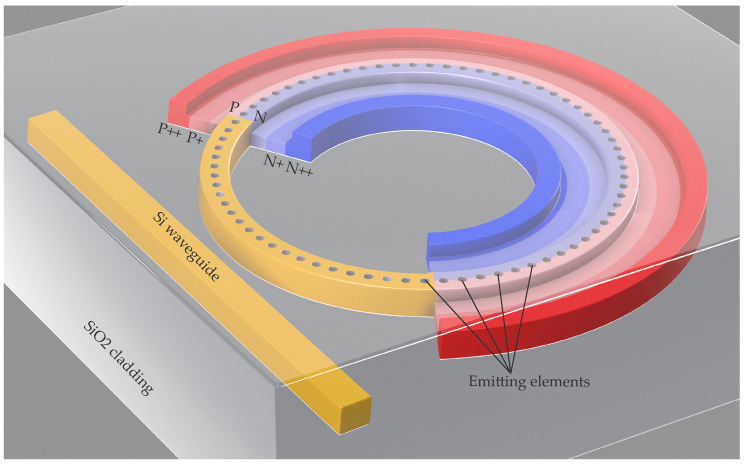
3D view of the proposed device. The pn-depletion diode follows the Si rib bent waveguide. The different shades of blue and red represent the different electron (blue) and hole (red) concentrations where darker shades correspond to higher concentrations.

**Figure 2:**
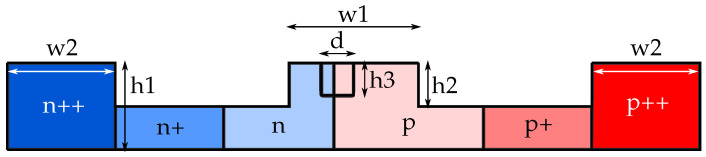
Cross-section of the doped part of the ring. N and P are for the electrons and holes concentrations, respectively: ++ is $10^{20}$ ${\mathrm{cm}}^{-3}$, + is $10^{19}$ ${\mathrm{cm}}^{-3}$, and without sign is $5\times 10^{18}$ ${\mathrm{cm}}^{-3}$. d and h3 denote the hole diameter and height, respectively.

A schematic view of the proposed device is depicted in Figure 1. The device consists of a straight input waveguide and a ring resonator with a light-emitting grating (etched holes on top of the ring waveguide) and a pn-depletion diode, imprinted over the part of the ring. The diode cross-section in detail is shown in Figure 2 and its dimensional quantities listed in the Table 1.

The main principle of vortex beam generation is similar to the presented in [37], where the order of the radiated OAM carrying beam *ℓ* satisfies the following condition:

(1) ℓ=p−gq,

where *p* is the WGM order in the ring, *q* is the number of grating elements in the ring resonator, and *g* is the diffraction order, which is an integer and can be calculated as [37]:

(2) $$\left(n_{eff}-1\right)\frac{2\pi R}{q\lambda }<g<\left(n_{eff}+1\right)\frac{2\pi R}{q\lambda },$$

where *R* is the ring resonator radius, $n_{eff}$ is the effective index of the ring waveguide, and λ is the operating wavelength.

To switch the emitted vortex order and the emitter resonance wavelength, it is necessary to change the effective index of the ring resonator. There are two main methods to modify the effective index: to use thermo-optical effect and electro-optical effect (mainly plasma dispersion effect). In the context of optical switches and modulators, the first effect is usually used in cases when GHz response frequencies are not required [36]. The main disadvantages of the thermo-optical effect for modulation and switching are milli- to microsecond response and mW order power consumption [38]. In contrast, the electro-optical effect is characterized by nanosecond to sub-nanosecond response and in most cases much lower, or at least comparable to the thermo-optical case, power consumption [36]. On the other hand, realizations of electro-optical effects impose higher losses and crosstalk [36], however, the advantage of quick response usually overrides these problems.

In our device, we propose to use an inversely-biased pn-depletion diode, integrated into the ring resonator, providing to change the refractive index of the ring waveguide due to the plasma dispersion effect [39].

To model the device, we used modified coefficients for the Soref and Bennet model, where a change in absorption coefficient and refractive index for wavelength 1.55 µm (C-band) can be expressed as [40]:

(3) $$\Delta \alpha =\Delta \alpha_{e}+\Delta \alpha_{h}=8.88\times 10^{-21}\Delta N_{e}^{1.167}+5.84\times 10^{-20}\Delta N_{h}^{1.109},$$

(4) $$-\Delta n=\Delta n_{e}+\Delta n_{h}=5.4\times 10^{-22}N_{e}^{1.011}+1.53\times 10^{-18}\Delta N_{h}^{0.838},$$

where $\Delta N_{e}$ and $\Delta N_{h}$ are the changes in number of electrons and holes in the active region, respectively.

The resonant wavelength $\lambda_{res}$ of the ring can be calculated as [41]:

(5) $$\lambda_{res}=\frac{n_{eff}L}{m},m=1,2,3,\ldots ,$$

where *L* is the circumference of the ring resonator.

The proposed device can be fabricated using a standard silicon-on-insulator (SOI) platform, or other platforms supporting doping, where the waveguides with pn-junction can be implemented. For our simulations, we considered a generic SOI platform with the Si layer thickness of 220 nm. The designed waveguide structures in this platform can be typically fabricated using 193 nm deep ultraviolet photolithography, or electron-beam lithography (EBL).

## 3. Simulation Results

**Figure 3:**
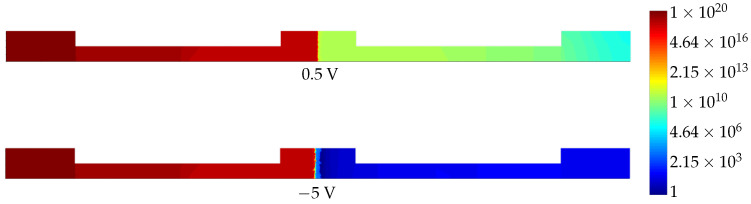
Distributions of electrons in the cross-section of the rib waveguide for different applied voltage values.

For the numerical modeling and simulation of the device, we used the Ansys Lumerical software. The first step was calculating the carrier number in the cross-section of the ring waveguide. This distribution was simulated in Lumerical Device and further exported into a mat-file for application in the following modeling steps. The obtained carrier number distributions are presented in Figure 3.

In the next step, we used the obtained carrier distributions to account for the effective index change due to the free plasma dispersion effect in Lumerical FDTD. To implement this, Lumerical’s silicon material model with the modified coefficients from Equation (4) have been applied. Next, we calculated the resonance characteristics of the microring emitter when voltages of 0.5 V and −5 V are applied between the inner and outer parts of the ring waveguide. Finally, we investigated the emitted field distributions (after spreading 4 µm from the device) at the resonance wavelengths of interest (near 1550 nm).

**Figure 4:**
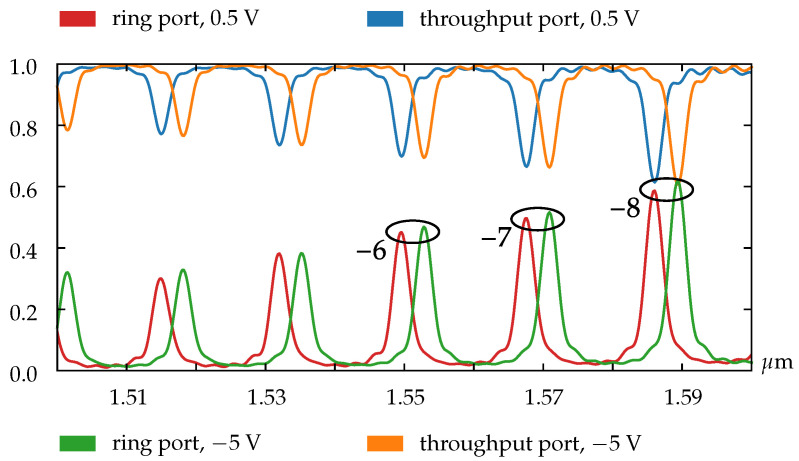
Normalized transmission spectra for the ring with radius 5.5 µm.

As our simulations show, for the ring resonators with radii of less than 25 µm there is no possibility to obtain the resonance shift equal or greater than the device free spectral range (FSR). The main reason is that the FSR value of the small rings is biggish, and the length of the doped region is too short to obtain the corresponding phase shift. This limitation does not allow us to realize the OAM order modulation for such small rings, but as shown in Figure 4 we can utilize pn-diode to adjust the resonance characteristics of the OAM emitter.

**Figure 5:**
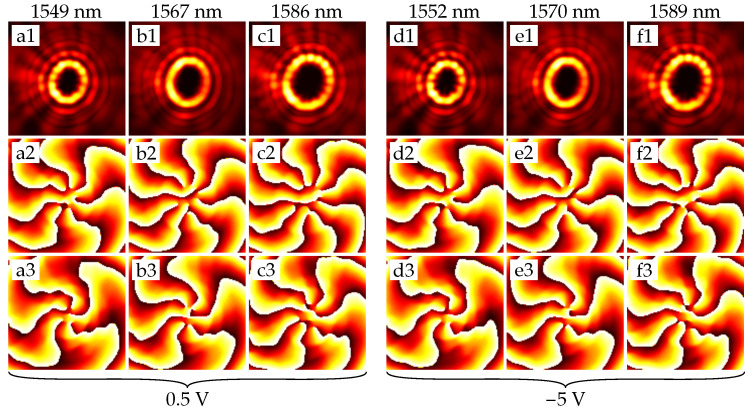
Intensity and phase distributions of the emitted fields from the ring with radius 5.5 µm. (**a1**–**a3**) is an intensity, $arg(E_{x})$ and $arg(E_{y})$ at a given resonant wavelength, respectively. Phase distributions are obtained after passing the field through the quarter-wave plate, so its *x*-component’s azimuthal order is above by one, and its *y*-component’s azimuthal order is below by one than the actual order of the generated vector vortex beam. Distribution patterns (**b1**–**b3**)–(**f1**–**f3**) are obtained similarly. (**a1**–**a3**), (**b1**–**b3**), and (**c1–c3**) refer to the resonances at the voltage of 0.5 V, and (**d1**–**d3**), (**e1**–**e3**), and (**f1**–**f3**) to the resonances at −5 V.

Figure 5 shows that the vortex order does not change within one FSR. This effect confirms that we have only functionality of the resonance adjustment for small rings.

**Figure 6:**
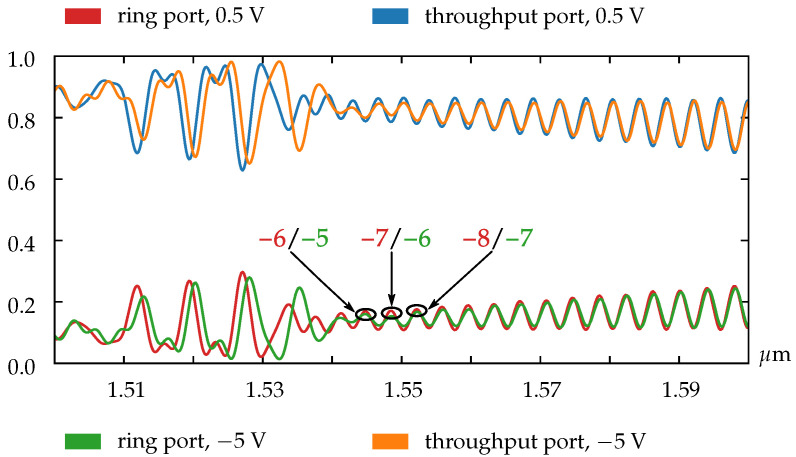
Normalized transmission spectra for the ring with radius 26.5 µm.

**Figure 7:**
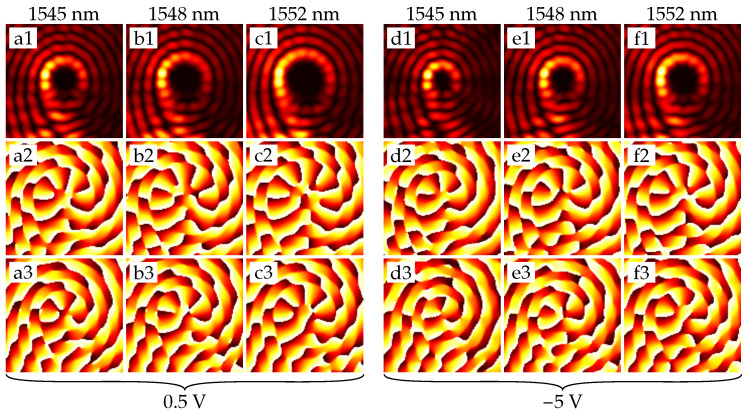
Intensity and phase distributions of the emitted fields from the ring with radius 26.5 µm. (**a1**–**a3**) is an intensity, $arg(E_{x})$ and $arg(E_{y})$ at a given resonant wavelength, respectively. Phase distributions are obtained after passing the field through the quarter-wave plate, so its *x*-component’s azimuthal order is above by one, and its *y*-component’s azimuthal order is below by one than the actual order of the generated vector vortex beam. Distribution patterns (**b1**–**b3**)–(**f1**–**f3**) are obtained similarly. (**a1**–**a3**), (**b1**–**b3**), and (**c1–c3**) refer to the resonances at the voltage of 0.5 V, and (**d1**–**d3**), (**e1**–**e3**), and (**f1**–**f3**) to the resonances at −5 V.

In the case of larger rings (especially for the rings with radii of greater than 25 µm), the FSR value becomes small enough to realize the change in the OAM state. As can be seen from Figure 6, the resonances shift over the one FSR value. Through this effect, the OAM order modulation of the optical signal can be obtained, as can be seen in Figure 7.

In addition, note that the resonant curves have smaller peaks due to the lower coupling in the case of the larger ring. The weakening of coupling can be explained by the increased complexity and hence the heterogeneity of the emitter, containing about 300 grating elements, which becomes difficult for optimizing due to doping. Nevertheless, we have shown that the proposed scheme generally is capable to implement the OAM order switching.

## 4. Analysis of the Emitted Field Propagation

**Figure 8:**
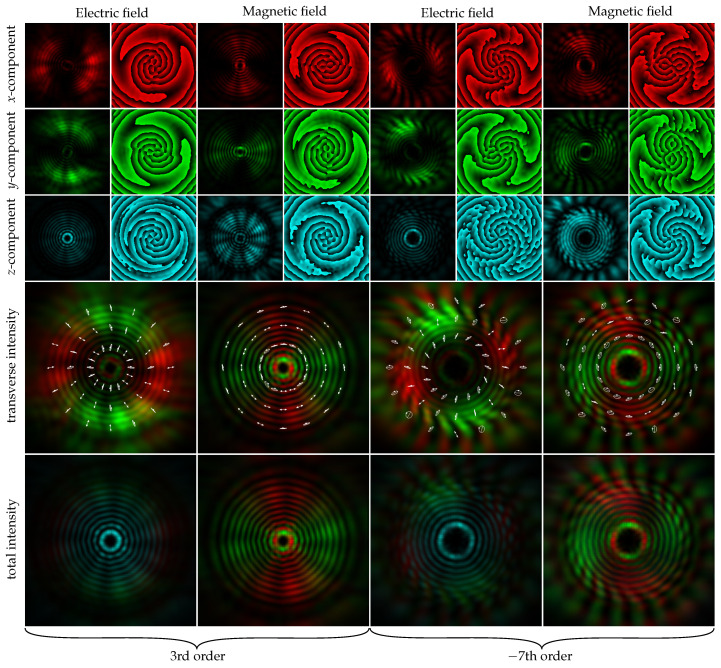
Distribution patterns of the emitted field in the near field. Size of the field plots is 26 µm × 26 µm.

To ensure that the resulting beams retain their vortex structure as they propagate through free space (for example, before injecting them into the fiber), we performed calculations using some obtained field distributions from the emitter. The near-field distributions for the vortices of 3rd (field 1) and 7th (field 2) orders are shown in Figure 8.

As it can be seen, field 1 is a radially polarized field with the 3rd order vortex phase component. Field 2 is a hybrid-polarized (superposition of radial and azimuthal polarization) field with a 7th order vortex phase component. There is comparably high intensity in the *E*-field component ${\left|E_{z}\right|}^{2}$ due to the diffraction in the near field [42,43,44].

Primarily, we calculated the electromagnetic field in the lens focal plane (focal length f=1.3 mm, numerical aperture of the lens NA=0.01) by using the transverse electric field components of the incident beam and the vector propagation operator [45,46,47]:

(6) $$\left[\begin{array}{c} E\left(r,\varphi ,z\right)\\ H\left(r,\varphi ,z\right) \end{array}\right]=-\frac{if}{\lambda }\int_{0}^{\theta_{\max}}\int_{0}^{2\pi }\left[\begin{array}{c} V_{E}\left(\theta ,\phi \right)\\ V_{H}\left(\theta ,\phi \right) \end{array}\right]\exp\left\{ik\left[r\sin\theta \cos\left(\phi -\varphi \right)+z\cos\theta \right]\right\}\sqrt{\cos\theta }\sin\theta d\theta d\phi$$

where $\sin\left(\theta_{\max}\right)$ corresponds to the lens numerical aperture, polarization vectors are defined on the transverse electric field components of the incident beam $E_{0x}\left(\theta ,\phi \right)$ and $E_{0y}\left(\theta ,\phi \right)$ applying the following equations:

(7) $$V_{E}\left(\theta ,\phi \right)=\left[\begin{array}{cc} A\left(\theta ,\phi \right) & C\left(\theta ,\phi \right)\\ C\left(\theta ,\phi \right) & B\left(\theta ,\phi \right)\\ -D\left(\theta ,\phi \right) & -E\left(\theta ,\phi \right) \end{array}\right]\left(\begin{array}{c} E_{0x}\left(\theta ,\phi \right)\\ E_{0y}\left(\theta ,\phi \right) \end{array}\right),$$

(8) $$V_{H}\left(\theta ,\phi \right)=\left[\begin{array}{cc} C\left(\theta ,\phi \right) & -A\left(\theta ,\phi \right)\\ B\left(\theta ,\phi \right) & -C\left(\theta ,\phi \right)\\ -E\left(\theta ,\phi \right) & D\left(\theta ,\phi \right) \end{array}\right]\left(\begin{array}{c} E_{0x}\left(\theta ,\phi \right)\\ E_{0y}\left(\theta ,\phi \right) \end{array}\right),$$

(9) $$\left\{\begin{array}{c} A\left(\theta ,\phi \right)=1-\cos^{2}\phi \left(1-\cos\theta \right),\\ B\left(\theta ,\phi \right)=1-\sin^{2}\phi \left(1-\cos\theta \right),\\ C\left(\theta ,\phi \right)=-\sin\phi \cos\phi \left(1-\cos\theta \right),\\ D\left(\theta ,\phi \right)=\cos\phi \sin\theta ,\\ E\left(\theta ,\phi \right)=\sin\phi \sin\theta . \end{array}\right.$$

**Figure 9:**
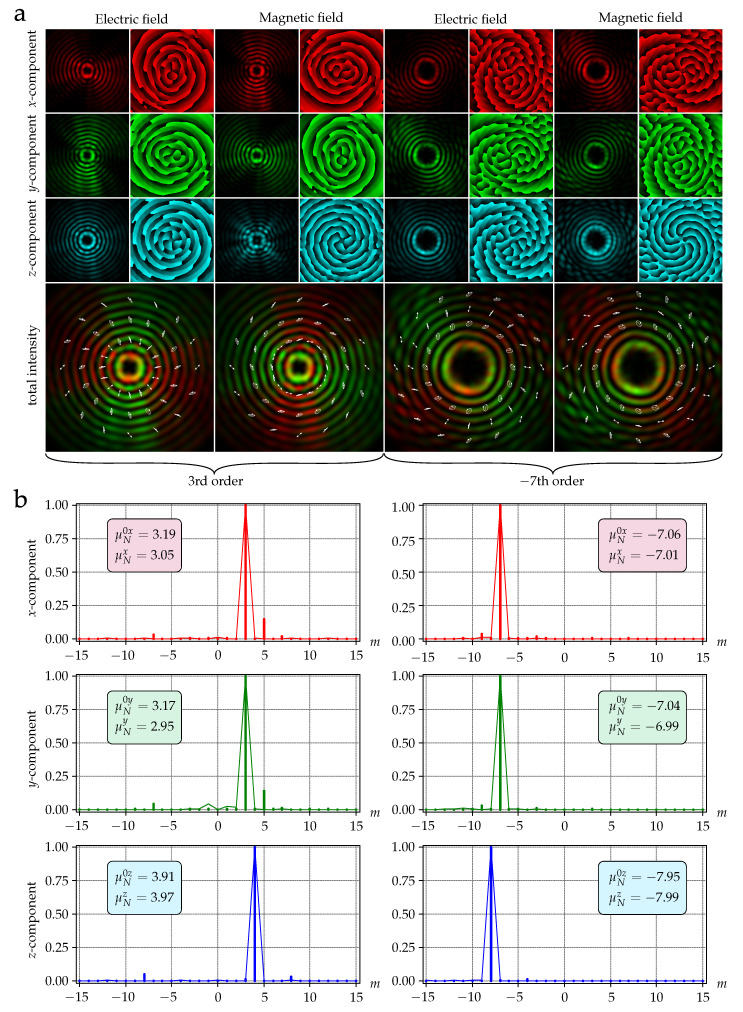
(**a**) Distribution patterns of the emitted fields in far-field. The size of the field plots is 2 mm × 2 mm. (**b**) Normalized intensities of the expansion coefficients in the basis of angular harmonics (OAM spectra) of the components of the electric field 1 (left column) and field 2 (right column): for the incident field ${\left|c_{m}^{0t}\right|}^{2}$ (vertical lines) and for far-field ${\left|c_{m}^{t}\right|}^{2}$ (envelopes).

The calculation results, corresponding to the field in the far zone, are shown in Figure 9. It can be seen that the beam structure has changed, becoming close to the radially polarized Bessel beam of the third order, but the phase and polarization states of the beam are preserved. Similarly, a hybrid-polarized seventh-order Bessel beam was formed in the far-field.

Figure 9 also shows the results of the decomposition of each component of the electric fields 1 and 2, based on angular harmonics for the incident field [48]:

(10) $$c_{m}^{0t}=\frac{1}{2\pi }\int_{0}^{\theta_{\max}}\int_{0}^{2\pi }E_{0t}\left(\theta ,\phi \right)\exp\left(-im\phi \right)d\theta d\phi ,$$

and in far field:

(11) $$c_{m}^{t}=\frac{1}{2\pi }\int_{0}^{R}\int_{0}^{2\pi }E_{t}\left(r,\varphi ,z_{f}\right)\exp\left(-im\varphi \right)rdrd\varphi ,$$

where t={x,y,z} and *m* is integer.

Using the coefficients from Equations (10) and (11), the OAM value for each field component can be calculated by the following formula [49]:

(12) $$\mu_{N}^{t}=\left(\sum_{m=-N}^{N}m{\left|c_{m}^{t}\right|}^{2}\right){\left(\sum_{m=-N}^{N}{\left|c_{m}^{t}\right|}^{2}\right)}^{-1}$$

where 2N+1 is the number of calculated decomposition coefficients (we used N=15).

As follows from the results shown in Figure 9, the value of OAM (12) is practically preserved in the far-field for all the field components of the vector vortex beams. In this case, the OAM value for the longitudinal component differs by one from the OAM values of the transverse components, which is in full agreement with the theory [44,45,46].

## 5. Discussion

In this paper, we proposed the novel design of the microring-based vortex beam emitter. First, the proposed scheme provides a possibility to realize the adjustment of the spectral properties of optical vortex emitter and, correspondingly, reduce the dependence of the emitter resonances on the fabrication errors. Also, for the structures with larger radii (starting from approximately 25 µm), it becomes possible to realize the OAM switching in the wavelength domain, which can be used for electro-optical OAM modulation of the signal.

As mentioned above, the proposed scheme is expected to provide much faster resonance adjustment or OAM order switching with much lower power consumption compared to the schemes based on thermo-optical effect following from the operating characteristics of the inversely biased pn-junction. Moreover, the proposed integrated scheme is much smaller than the conventional discrete optics for generating optical vortices in free space. Generally, our device can be useful for future data transmission systems with spatial division (de)multiplexing, for OAM encoding, or in sensing systems.

It is worth noting, that tunable vortex mode emitter can be especially useful in applications where the beam’s OAM order and fine-tuning of its spectral characteristics are of decisive importance. For example, FBG temperature sensors, which are based on the interaction of OAM radiation with the transmission medium, are influenced by the measured external parameters. Theoretically, our proposed device can be used for spin [50] and lateral motion [51] detecting schemes. These methods are based on the light-matter interaction, which couples OAM and mechanical momentum. Also, it would be interesting to use such an emitter in an OAM-controlled hybrid plasmonic circuit for optical logic operations [52] and in other photonic circuits for information processing.

For further development of the proposed device, the metal mirror placed in the buried oxide [53] can be applied to suppress the cylindrical vector Bessel modes of higher orders, which are formed sideways, and therefore improve the emission efficiency. There is also a possibility to further optimize the coupling between the bus waveguide and the ring resonator to increase the depth of the resonances and raise the emitted vortex beam power.

## 6. Conclusions

In summary, we showed that by integrating a pn-diode in the ring waveguide of the OAM emitter, the carrier number can be controlled enabling a refractive index modulation, that allows rapidly changing the transmission spectrum of the emitter. This effect allows adjusting the emitter resonance to the desired wavelength while maintaining the required OAM in the case of rings with radii smaller than 25 µm. For the larger ring radii, it is possible to implement the OAM (de)multiplexing or modulation.

Modeling results have shown that our device in case of the small ring radius (5.5 µm) emits an optical vortex with the topological charge ℓ=−6 at the wavelength of 1549 nm and the voltage level of 0.5 V, and with the same azimuthal order at 1552 nm and −5 V. This provides a resonance wavelength shift while maintaining the required topological charge of the emitted vortex beam. In the case of the larger ring (with the radius of 26.5 µm) the voltage change provides the change in the topological charge of the emitted vortex beam (−7 and −6 at the wavelength of 1548 nm for the voltage levels of 0.5 V and −5 V, respectively). In a detailed study of the available beam distributions, we also ensured that the radiated beams maintain their topological charges while propagating in free space.

We believe that the presented results will be useful both for the further development of OAM-powered devices and for moving towards full-fledged photonic integration.

## Figures and Tables

**Table 1:** List of geometric values in Figure 2.

Dimension	w1	w2	h1	h2	h3	d
Value	0.545	0.5	0.22	0.11	0.07	0.15

## Data Availability

Not applicable.
